# The Effect of Laser Re-Solidification on Microstructure and Photo-Electrochemical Properties of Fe-Decorated TiO_2_ Nanotubes

**DOI:** 10.3390/ma13184019

**Published:** 2020-09-10

**Authors:** Piotr Kupracz, Katarzyna Grochowska, Jakub Karczewski, Jakub Wawrzyniak, Katarzyna Siuzdak

**Affiliations:** 1 Centre of Plasma and Laser Engineering, The Szewalski Institute of Fluid-Flow Machinery PASci, Fiszera 14 Street, 80-231 Gdańsk, Poland; jwawrzyniak@imp.gda.pl (J.W.); ksiuzdak@imp.gda.pl (K.S.); 2 Faculty of Applied Physics and Mathematics, Gdańsk University of Technology, Narutowicza 11/12 Street, 80-233 Gdańsk, Poland; jakub.karczewski@pg.edu.pl

**Keywords:** TiO_2_ nanotubes, Fe_2_O_3_, iron oxide nanoparticles, water splitting, laser processing

## Abstract

Fossil fuels became increasingly unpleasant energy source due to their negative impact on the environment; thus, attractiveness of renewable, and especially solar energy, is growing worldwide. Among others, the research is focused on smart combination of simple compounds towards formation of the photoactive materials. Following that, our work concerns the optimized manipulation of laser light coupled with the iron sputtering to transform titania that is mostly UV-active, as well as exhibiting poor oxygen evolution reaction to the material responding to solar light, and that can be further used in water splitting process. The preparation route of the material was based on anodization providing well organized system of nanotubes, while magnetron sputtering ensures formation of thin iron films. The last step covering pulsed laser treatment of 355 nm wavelength significantly changes the material morphology and structure, inducing partial melting and formation of oxygen vacancies in the elementary cell. Depending on the applied fluence, anatase, rutile, and hematite phases were recognized in the final product. The formation of a re-solidified layer on the surface of the nanotubes, in which thickness depends on the laser fluence, was shown by microstructure studies. Although a drastic decrement of light absorption was recorded especially in UV range, laser-annealed samples have shown activity under visible light even 20 times higher than bare titania. Electrochemical analysis has shown that the improvement of photoresponse originates mainly from over an order of magnitude higher charge carrier density as revealed by Mott-Schottky analysis. The results show that intense laser light can modulate the semiconductor properties significantly and can be considered as a promising tool towards activation of initially inactive material for the visible light harvesting.

## 1. Introduction

The increasing global energy from renewable sources demand of well-developed society conflicts with the structure of the world energy balance, where fossil fuels take over 80% [1]. The most promising candidate for the modern energy source is solar energy, as it can be easily converted into electric power almost everywhere. However, the fluctuant insolation and limited electric energy storage technology make its application unsuitable for long-range transport and long-term storage. Photo-generation of H_2_ from water seems to be one of very attractive technologies of solar power accumulation due to easiness of chemical to electrical energy conversion [2]. Unfortunately, the efficiency of the solar enhanced water splitting systems suffers from the lack of low-cost and highly effective electrode materials. Currently, two main material classes are considered to be the most suitable namely nanoscale noble metals and semiconductors. Since the former is very expensive, the researchers focus on photoactive semiconductors [3]. For example, the perfect photo-anode devoted to the water splitting system should have direct optical bandgap around 2.2 eV, low overpotential in reference to oxygen or hydrogen evolution reaction, and low recombination rate of charge carriers [4,5]. In the case of the photoactive anode material, semiconductors like RuO_2_, Pb_3_O_4_, and CdS are the nearest to satisfying these requirements; however, CdS undergoes corrosion during exposition to light, and utilization of Pb and Cd is limited due to their toxicity, while Ru is very expensive [6]. On the other hand, ZnO_2_, TiO_2_, BaTiO_2_, and SnO_2_ are generally regarded as non-toxic, chemically inert, and inexpensive but exhibit higher electron-hole recombination rate and much wider bandgap (3.0–3.5 eV) than RuO_2_, Pb_3_O_4_, and CdS [7,8], which limits their effective light-gathering ability to less than 6% of solar spectrum power. Nevertheless, it should be realized that free zinc oxide or titania nanoparticles can negatively impact the DNA chains and proteins [9,10], but formation of the material already fixed onto the stable substrate and their further usage in the immobilized form minimizes this risk.

Fujishima and Honda [11], as a first, focused world attention on the photo-electrochemical properties of TiO_2_ building the first solar-driven electrochemical cell. However, works on its application in the water-splitting system have accelerated after the successful dye sensitization of TiO_2_, which extends light absorption to the visible part of the spectrum [12]. These two seminal works became the basis for the research on a wide branch of modified TiO_2_ for electrodes in water-splitting systems.

Since the recombination rate of photo-generated minority carriers depends on the diffusion path length to the electrode surface, where they can take part in a water molecule oxidation [13], the structural engineering is especially focused in a way to produce highly porous structures. As it has been already shown [14,15,16,17,18,19,20], the preparation of TiO_2_ in a form of various nanostructures, like nanobelts, nanorods or nanotubes drastically improves photo-electrodes performance in a reference to the plane material. However, its tubular structure synthesized by anodization is most frequently examined due to perpendicular orientation of hollow cylinders regarding the metal substrate reducing the charge recombination and the scalability of the fabrication process [15,21]. Additionally, the geometrical parameters of titania nanotubes (TiO_2_-NTs) could be easily optimized during the synthesis, giving rise to the specific surface and light absorption of the resulting material [14,22]. The advantage of the direct contact between the electrode material and the current collector, as well as well-developed surface area, has been used in prototype supercapacitors when the further hydrogenation via cathodic polarization or plasma treatment in hydrogen atmosphere is applied. Further improvement of TiO_2_-NTs electrochemical performance could be achieved by the formation of junction between TiO_2_ and covering semiconductors with higher positioned valence and conduction bands which promotes dissociation of photo-generated electron-hole pair [23]. Such an approach has more advantages since the presence of well-adjusted semiconductor on TiO_2_-NTs can decrease the Shottky barrier height at the electrode-electrolyte interface [6,15] and extends the range of the heterojunction photoresponse from ultraviolet (UV) to the visible region. Recently, the usage of transition metal oxides, like NiO, Fe_2_O_3_, or Cu_2_O [6,24,25,26,27], has been extensively explored as the decoration can be achieved via various methods, like chemical deposition [28], electrochemical deposition [29], alternative layer deposition [30], hydrothermal deposition [23], or photodecomposition [31]. Among others, the TiO_2_-α-Fe_2_O_3_ heterojunction is the most explored due to the well-suited for light gathering bandgap (2.2 eV) of the latter, its low cost, and a relative misalignment of the semiconductors band edges promoting holes and electrons accumulation in TiO_2_ and α-Fe_2_O_3_, respectively [24,32,33,34]. Further junction enhancement is observed when pseudobrookite (TiO_5_) is formed at the interface between hematite and titania [34,35,36]. Due to its narrower bandgap (2.1 eV) and alignment of valence and conduction bands, the TiO_2_-Fe_2_TiO_5_-α-Fe_2_O_3_ heterojunction additionally facilitates solar absorption and promotes the charge carriers’ separation.

Apart from the modification by other metal oxides, another strategies to improve the performance of pristine TiO_2_ based electrode cover, e.g., treatment with plasma, annealing in hydrogen, and laser irradiation [37]. Among others, the ns laser pulse induces fast heating and cooling process, even above the melting point, therefore, its interaction with multi-layer composite may result in diffusion of atoms across the interphase, as well as the formation of metastable phases and structural defects [38]. That technique is extremely fast, is easy-scalable for large areas, and due to a wide range of processing parameters can be applied to various materials [39]. The modification of anatase TiO_2_-NTs electrode using Nd:YAG laser was shown to enhance charge carrier donor density over one order of magnitude and a shift of the flat-band potential up to +0.7 V vs. Ag|AgCl|0.1 M KCl [16,39]. However, the modification leads to the photo-response improvement by no more than a few tens of percent. On the other hand, doping TiO_2_ by Fe decreases recombination of the photogenerated charge carriers, and provides narrower bandgap [40,41,42], while charge carriers density in Ti-doped hematite is one order of magnitude higher in reference to the pristine [35]. Although the coverage of TiO_2_ by Fe_2_O_3_, and doping TiO_2_ with Fe, as well as Fe_2_O_3_ with Ti, and laser treatment of TiO_2_-NTs lead to the improvement on the electrode performance, the combined approach has not been examined till now.

In this work, an array of parallel TiO_2_-NTs were electrochemically fabricated and covered by several nm thick Fe layer. As obtained hetero-structures were further modified by the pulsed Nd:YAG laser treatment, followed by the calcination in an electrical furnace. The main attention was paid to the impact of metal layer thickness, and laser fluence on the electrochemical performance of Fe-decorated TiO_2_-NTs samples. Surface morphology and composition analyses were performed using scanning electron microscopy, Raman spectroscopy, X-ray diffraction, and X-ray photoelectron spectroscopy. The diffuse reflectance spectroscopy was employed to study the light absorption capability and optical band-gap position. Electrochemical measurements have proven that easily scalable laser processing over the titania NTs with sputtered thin iron film can significantly change the electric properties, namely resulting in the cathodic shift of the flat-band potential and growth of negative charge carriers density, which inflicts 20-times enhancement of the charge carriers photo-generation efficiency under visible light irradiation compared to the pristine TiO_2_-NTs.

## 2. Materials and Methods

**Figure 1:**
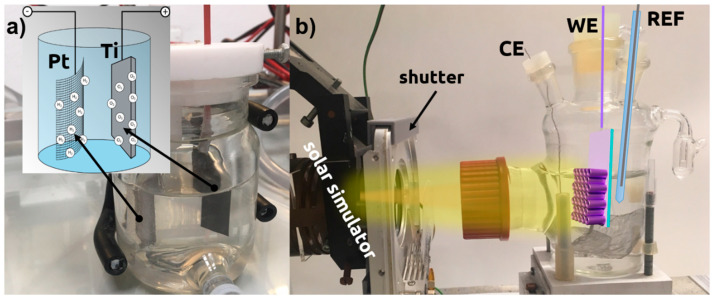
The experimental setups used for (**a**) the anodization and (**b**) photoelectrochemical characterization.

Highly-oriented titanium nanotubes arrays were produced by one-step electrochemical anodization in a two-electrode arrangement. The titanium foil (99.7%, Strem) with dimensions of 2 × 3 cm^2^ acted as an anode and Pt rectangular mesh as a cathode in the electrolyte consisted of 0.27 M NH_4_F (p.a. Chempur) and 15 wt% of deionized water in ethylene glycol (p.a. Chempur), similar to other treatment procedures [43]. The electrolyte temperature was kept at 23 °C with the thermostat (Julabo F-12, Julabo, Seelbach, Germany). The experimental setup used for anodization is shown in Figure 1a. Ti foil was cleaned by a three-step process, i.e., firstly the 10-min ultrasonic bath in acetone (Protolab, Słupsk, Poland), next in ethanol (96%, Chempur, Piekary Śląskie, Poland), and finally in deionized water (0.08 μS, Hydrolab HLP-5p, Hydrolab, Straszyn, Poland). Acetone and ethanol are used for degreasing and removing of the organic contamination while final water treatment simply removes the residues of acetone and ethanol because in the main anodization bath those compounds are not present. After all, samples were rinsed with isopropanol (p.a. POCH) and left to dry in the air. A detailed description of the anodization process can be found in Reference [26]. The titanium foil with as-growth TiO_2_-NTs was annealed in an electrical furnace (Nabertherm, Lilienthal, Germany) in the static air with a heating rate of 2 °C/min up to 450 °C, kept 2 h, and cooled freely. Next, TiO_2_-NTs serve as a support for iron deposition utilizing magnetron sputtering (Q150S, Quorum Technologies, Lewes, UK) with mounted highly pure Fe target (99.5 % EM-Tec). The Fe thickness was controlled by quartz microbalance and implemented program and set to 5, 10, and 15 nm.

Next, the pulsed laser modification of Fe-decorated TiO_2_-NTs was carried out using a Q-switched Nd:YAG (Quantel, Lannion, France) pulse laser generating 6 ns pulses at the wavelength of 355 nm with the repetition rate of 2 Hz. The laser beam went through a square homogenizer and 200-mm focal length lens giving a 2.7-mm wide square spot of uniform irradiance. In order to increase the uniformity and size of the modified area to ~30 mm^2^, the samples were moved back and forth in accordance to laser beam with a constant speed of 3.7 mm/min during the modification. The surface of samples was laser-annealed with an energy fluence (F) of 40, 80, 120, 160, 200 mJ/cm^2^ under the vacuum of 3 × 10^−5^ mbar. After all, samples calcination was repeated maintaining the same parameters. As obtained samples are further referred as FeXLY, where X is the Fe thickness, and Y is the laser fluence applied during the annealing.

The samples’ morphology was examined with Scanning Electron Microscope FE-SEM, FEI Quanta FEG 250 (Thermo Fisher, Waltham, MA, USA) with a secondary electron detector maintaining 10 kV accelerating voltage. The X-Ray Photoelectron Spectroscopy studies were carried out in the Ti2p, Fe2p, O1s, and C1s binding energy range using Escalab 250Xi spectroscope (Thermo Fisher, Waltham, MA, USA). The spectroscope was equipped in Al Kα monochromatic X-ray source (spot diameter 650 µm), operating at 20 eV pass energy. Charge compensation was provided utilizing a flood gun, with the final calibration of the obtained spectra based on the signal derived from adventitious carbon (C1s at 284.6 eV) [44]. The structure analysis was carried out by X-ray diffractometry (XRD) over the range of 20°–90° with a Bruker D2Phaser (Bruker, Billerica, MA, USA) diffractometer with CuKα radiation, equipped with a XE-T detector, and the Raman spectroscopy using a confocal micro-Raman spectrometer (InVia, Renishaw, Wotton-under-Edge, Gloucestershire, UK) with an argon-ion laser source emitting at 514 nm and operating with the power of 5 mW. Reflectance spectra were taken by diffusive reflectance spectroscopy with PerkinElmer Lambda 35 (PerkinElmer, Waltham, MA, USA) dual-beam spectrophotometer in the range of 200–900 nm with a scanning speed of 120 nm/min.

The electrochemical properties were measured in a three-electrode system with Autolab PGStat 302 N potentiostat-galvanostat Metrohm (Autolab, Utrecht, Holland) system, where Pt mesh and Ag|AgCl|0.1 M KCl served as a counter (CE) and the reference electrode (REF), respectively. The investigated material was used as working electrode (WE). The 0.5 M NaOH (p.a. POCH) electrolyte was initially deaerated with argon (5N, Air Liquide) and separated from oxygen by Ar continuous flow. The sun-simulator built of 150 W Xenon lamp (Optel, Opole, Poland) equipped with the AM 1.5 filter and additional mobile GG420 UV filter (Schott, Mainz, Germany) was used to perform photo-electrochemical measurements under the light of full solar light and limited to visible spectra, respectively. The whole experimental setup dedicated for photoresponse investigations is presented in Figure 1b.

Cyclic voltammetry (CV), as well as linear voltammetry (LV) measurements, were carried out in the potential range from −1.1 V to +0.9 V vs. Ag|AgCl|0.1 M KCl, with the scan rate of 50 mV/s and 10 mV/s, respectively. The chronoamperometry (CA) under varying light conditions was performed with the working electrode polarization set to +0.4 V vs. Ag|AgCl|0.1 M KCl. The electrochemical impedance for the Mott-Schottky analysis was measured at a single frequency of 1 kHz with a 10-mV amplitude of the sinusoidal signal and in the potential range from −1.0 to +0.8 V versus Ag|AgCl|0.1 M KCl. The stabilization time of 30 s before each impedance record was applied to reduce the influence of charging/discharging processes. The flat-band potential (*E_FB_*) and majority charge carriers density (*N_d_*) were calculated according to the Mott-Schottky Equation (1) [45]:

(1) $$\frac{1}{C_{SC}^{2}}=\frac{2}{\varepsilon_{0}\varepsilon_{R}AeN_{d}}\left(E_{FB}+E+k_{B}T\right),$$

where *C_SC_* is the capacitance of the space charge layer, *E* is bias potential, *ε_0_* and *ε_R_* are the vacuum and TiO_2_ permittivity, and *e* stays for the elementary charge. For the calculations, the following values were taken: *ε_R_* = 38 [46] for TiO_2_ (anatase), *ε*_0_ = 8.85 × 10^−12^ F/m, and *e* = 1.602 × 10^−19^ C.

## 3. Results and Discussion

**Figure 2:**
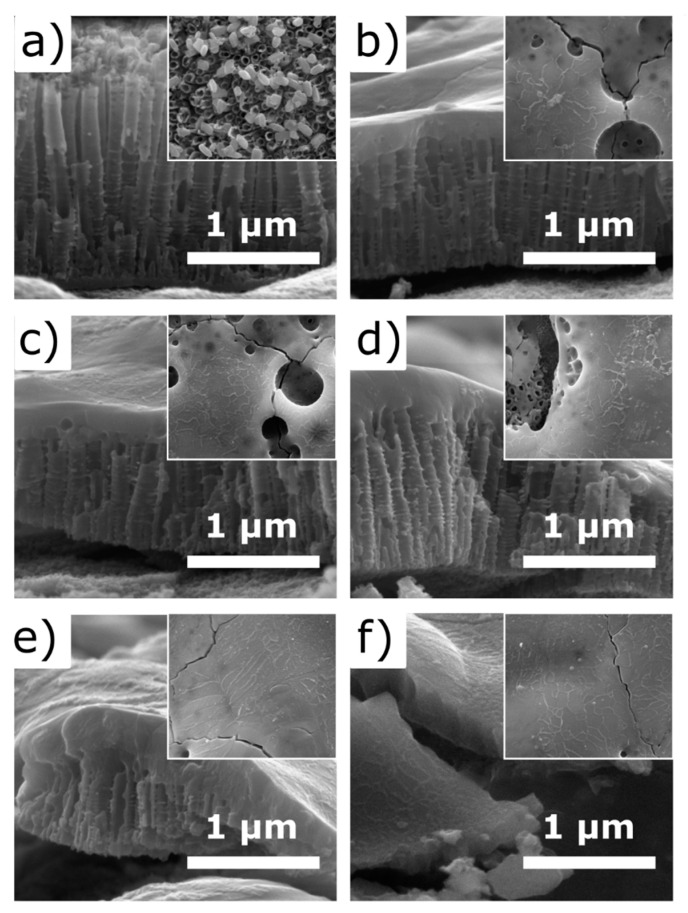
SEM images of laser-treated titania nanotubes (TiO_2_-NTs) covered by 10 nm Fe film from top (inset) and side. Images show the samples (**a**) untreated and treated by laser of (**b**) 40 mJ/cm^2^, (**c**) 80 mJ/cm^2^, (**d**) 120 mJ/cm^2^, (**e**) 160 mJ/cm^2^, and (**f**) 200 mJ/cm^2^ fluence.

**Figure 3:**
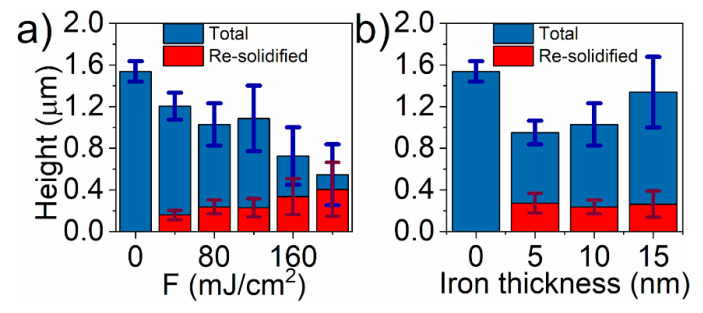
The dependence of the length of titania nanotubes (**a**) with 10 nm deposited Fe on the applied laser fluence and (**b**) on the iron layer thickness when the applied laser fluence equals 80 mJ/cm^2^.

The microstructure of TiO_2_-NTs covered by 10 nm of Fe and samples after the laser treatment of 355 nm wavelength is shown in Figure 2. The TiO_2_-NTs height, diameter, wall thickness, and distance between nanotubes were measured to be ca. 1500 nm, 117 nm, 17 nm, and 150 nm, respectively. The laser treatment with a fluence as low as 40 mJ/cm^2^ (Figure 2b) leads to the partial transformation of the TiO_2_-NTs surface to the 160 nm thick porous layer and in consequence to nanotubes height reduction (Figure 3). As the higher fluence of laser beam was applied (Figure 2c–f), a stronger shortening is observed until almost complete degradation of the well-ordered titania construction occurs for F = 200 mJ/cm^2^. The re-solidified surface layer porosity decrement accompanies that phenomenon, and its thickness grows from 160 nm to 405 nm as laser fluence increases from 40 mJ/cm^2^ to 200 mJ/cm^2^.

The effect of laser annealing on the pristine TiO_2_-NTs was studied by several authors [16,17,37,39,47]. In all cases, it was concluded that the laser fluence required to create the continuous re-solidified layer has to be higher than 40 mJ/cm^2^. Additionally, they have shown that, using that fluence, the TiO_2_-NTs rather join with the neighbors, creating stuck islands, than forming the compact thick layer. Comparing their results with the morphology of Fe-decorated TiO_2_-NTs, one may conclude that the presence of Fe plays an important role in the re-solidifying process. The absorption coefficients of Fe, Fe_2_O_3_, Fe_3_O_4_, and TiO_2_ for light of 355 nm wavelength are 1.15 × 10^6^ cm^−1^ [48], 3.95 × 10^5^ cm^−1^, 3.95 × 10^4^ cm^−1^ [49], and 4.12 × 10^3^ cm^−1^ [50], respectively. Since iron and iron oxides absorb the incident laser orders of magnitude stronger than TiO_2_, it becomes clear that the same laser fluence induces more efficient heating at the surface of Fe-decorated TiO_2_-NTs than bare ones leading to its more uniform re-solidification; please see work of Haryński et al. [39] for reference.

The dependency between TiO_2_-NTs length and the re-solidified layer is shown in Figure 3. As could be seen, the height of TiO_2_-NTs and the thickness of the re-solidified layer change almost linearly with the laser fluence from 1500 nm to 550 nm and from 0 nm to 410 nm, respectively. Taking into account their closed packed microstructure, their effective volume fill factor can be calculated by Equation (2):

(2) $$V_{\mathrm{fill}}=\;\frac{a^{2}\sqrt{3}}{2\pi \left(R^{2}-r^{2}\right)},$$

where a, R, and r, are separation, external and internal diameter of TiO_2_-NTs, respectively. Substituting the dimensions of bare TiO_2_-NTs from Figure 2, V_fill_ equals to ca. 0.45. Since the re-solidified layer in SEM images seems to be almost completely devoid of bubbles, the total volume of the forming layer should be nearly two times lower than unmodified TiO_2_-NTs. That explanation perfectly matches the registered evolution of TiO_2_-NTs morphology under the laser treatment (Figure 3a). On the other hand, the increasing thickness of the iron film on the sample treated with 80 mJ/cm^2^ fluence causes less intensive TiO_2_-NTs reduction under laser irradiation, while the measured thickness of the re-solidified layer remains almost constant (Figure 3b). It means that the porosity of re-solidified layer depends on the amount of Fe onto titania support. As shown above, stronger laser fluence leads to the less porous re-solidified layer. Therefore, the higher amount of Fe, the weaker heating takes place. The reflectivity measured for bulk Fe, Fe_2_O_3_, Fe_3_O_4_, and TiO_2_ for light of 355 nm wavelength is 0.68 [48], 0.25, 0.17 [49], and 0.25 [50], respectively. Thus, presence of pure iron may reduce the effectiveness of the laser annealing. It leads to the conclusion that the Fe-layer may play an opposite role. The small amount of Fe could increase the light absorption, leading to the uniform melting of the surface, while the thicker layer reflects the 355 nm radiation, reducing the effect of the laser annealing on the microstructure.

**Figure 4:**
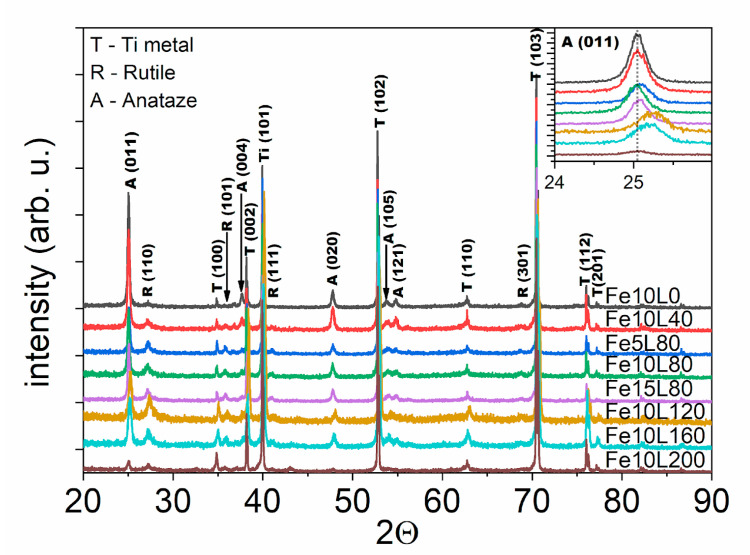
X-ray diffraction patterns of laser-annealed Fe-decorated TiO_2_-NTs.

X-ray diffraction patterns of modified TiO_2_-NTs are shown in Figure 4. Three major phases were recognized in the pattern, namely metallic Ti substrate with assigned peaks at 34.8°, 38°, 40°, 53°, 63° and 70° (PDF-2 ICDD: 00-044-1294), anatase TiO_2_: 25°, 37.8°, 48°, 53.7°, and 55° (96-900-8214), and rutile TiO_2_: 27°, 36°, 41°, and 69° (96-900-9084). Any other peaks which may reflect the presence of Fe, FeO, Fe_3_O_4_, and Fe_2_O_3_ are not seen. It is worthy of note that the shift towards higher angles is observed for the position of the peak at 25° (inset Figure 4), and, for the Fe10L40, Fe5L80, Fe10L80, Fe15L80, Fe10L120, and Fe10L160 samples, Δ2Θ is 0.05°, 0.08°, 0.05°, 0.04°, 0.21°, and 0.19°, respectively. As seen, the shift induced by the laser annealing increases with the fluence and reaches the maximum for 120 mJ/cm^2^. On the other hand, growing iron thickness reduces the structural changes induced by the treatment. In general, a positive shift of the diffraction pattern originates from the shrinkage of a primitive cell. Since TiO_2_-NTs were annealed in a presence of iron in a vacuum, it is possible that crystallographic cell shrinkage is induced by the oxygen escape from TiO_2_ lattice with an accompanying reduction of Ti^4+^ to Ti^3+^ [51] or by an additional substitution of Ti^3+^ by smaller Fe^3+^ ions [42].

**Figure 5:**
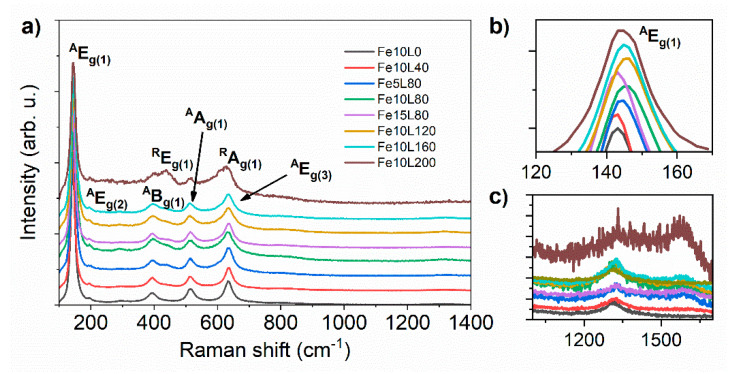
(**a**) Raman shift spectra recorded for laser-modified Fe-decorated TiO_2_-NTs. (**b**) The magnification of the anatase E_g(1)_. (**c**) Magnon scattering peaks.

The crystal structure of the laser-annealed Fe-decorated TiO_2_-NTs samples was distinguished by the Raman spectroscopy. As shown in Figure 5, five main peaks at 144, 196, 395, 515, and 634 cm^−1^ can be assigned to anatase E_g(1)_, E_g(2)_, B_g(1)_, A_g(1)_, and E_g(3)_ phonon modes, respectively [52]. However, with an increasing laser fluence from 40 to 200 mJ/cm^2^, the relative intensity of the anatase signal drops and peaks at 447 and 612 cm^−1^ originating from rutile E_g(1)_ and A_g(1)_ modes [53] become observable. Additionally, weak peaks at 1320 and 1576 cm^−1^ are seen (Figure 5c). The former arises from the interaction of two magnons created on antiparallel close spin sites [54] or from a second-order tone of a forbidden phonon mode [55], while the latter is ascribed to free magnon scattering [55]. Raman bands for hematite and magnetite expected at 550 and 660 cm^−1^ and at 193, 306, 538, and 668 cm^−1^, respectively [54,56], were not recorded due to their overlapping with stronger bands originated from anatase and rutile.

The variation of the relative intensity of the α-Fe_2_O_3_ and rutile-TiO_2_ compared to the anatase-TiO_2_ peaks imply that the laser annealing reduces the amount of the crystalline phase. Additionally, signal positions corresponding to main E_g(1)_ active anatase mode of laser-annealed samples depend on the utilized energy fluence during laser modifications; E_g(1)_ is located at 144.4, 143.8, 146.5, 146.5, 145.8 and 144.9 cm^−1^ for 0, 40, 80, 120, 160, and 200 mJ/cm^2^ energy fluence, respectively (Figure 5b). A blue shift of Raman peaks is usually referred to the presence of oxygen vacancies originating from distortion of crystal structure in defective TiO_2_ [57] or by substitution of Ti^4+^ by Fe^3+^ ions [58]. Thus, samples treated with 80 mJ/cm^2^ and 120 mJ/cm^2^ are expected to have the highest oxygen vacancies concentration. The strongest positive Raman and XRD shifts were observed for the Fe10L80 sample. In order to examine the influence of the iron layer thickness on the modified material structure, two samples, namely Fe5L80 and Fe15L80, were studied. However, Raman spectroscopy, as well as XRD pattern, have shown that Fe10L80 undergoes stronger structural evolution due to laser annealing; thus, further examinations have been limited to the sample covered by 10 nm of Fe.

**Figure 6:**
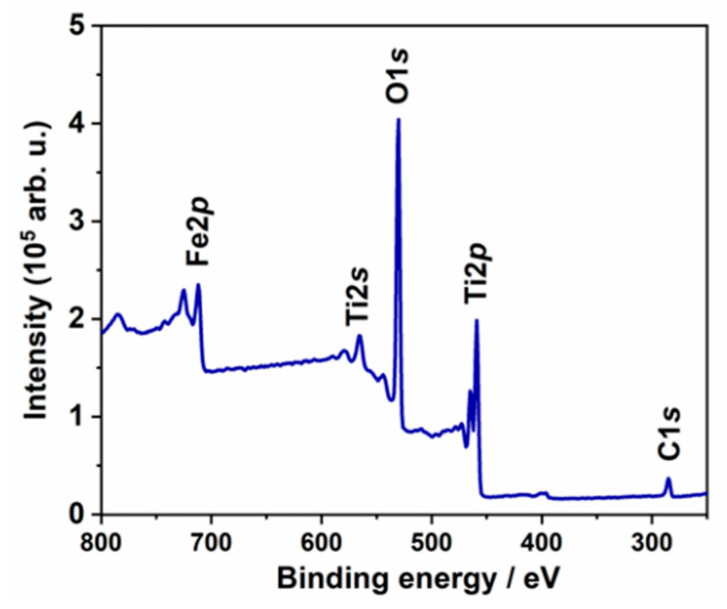
The XPS survey spectrum recorded for Fe10L80 sample.

The high-resolution XPS analysis allows providing insight into the surface chemistry of the investigated Fe80L10 sample. In Figure 6, the survey spectrum of the material is presented, where signals attributed to titanium, oxygen, iron, and carbon are indicated. Following that the high-resolution spectra were recorded and analyzed.

**Figure 7:**
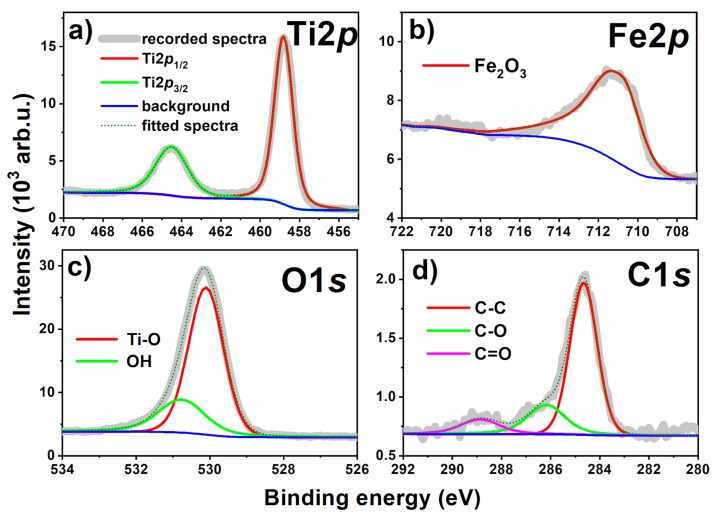
The XPS high resolution spectra registered for Fe10L80 sample: (**a**) titanium, (**b**) iron, (**c**) oxygen, and (**d**) carbon.

The Ti2p spectra are composed of a single chemical state, represented by the peak doublet where Ti2p_3/2_ is positioned at 458.8 eV (Figure 7a). The peaks in the deconvoluted energy range are characteristic for the TiO_2_ nanotubes as reported in our previous studies [39,59]. Although XRD and Raman spectroscopy may suggest the presence of Ti^3+^ ions, no other oxidation states were present confirming that the chemistry of TiO_2_-NTs was not significantly altered during the modification process. It supports the previous assumption that the structural changes may be caused by substitution of Ti^4+^ by Fe^3+^ ions. On the other hand, the broad spectra recorded in the Fe2p energy range is peaking at 710.9 eV (Figure 7b). The position of Fe2p_3/2_ peak, as well as complex multiplet-split peak shape, reveals its origin as Fe_2_O_3_. This observation is further confirmed by Fe^3+^ satellite visible at approx. 720 eV [60,61]. These findings are in good agreement with the nature of O1s components (see Figure 7c), two of which were identified and deconvoluted within this model. The primary component, at 530.1 eV, is characteristic for both TiO_2_ and Fe_2_O_3_. The second and smaller component could be attributed to surface hydroxyl species but also adventitious carbon contamination [39,44]. According to the surface area, the atomic share of particular elements has been determined: Ti: 18.3%, C: 11.3 %, O: 66.8%, and Fe: 3.6%.

**Figure 8:**
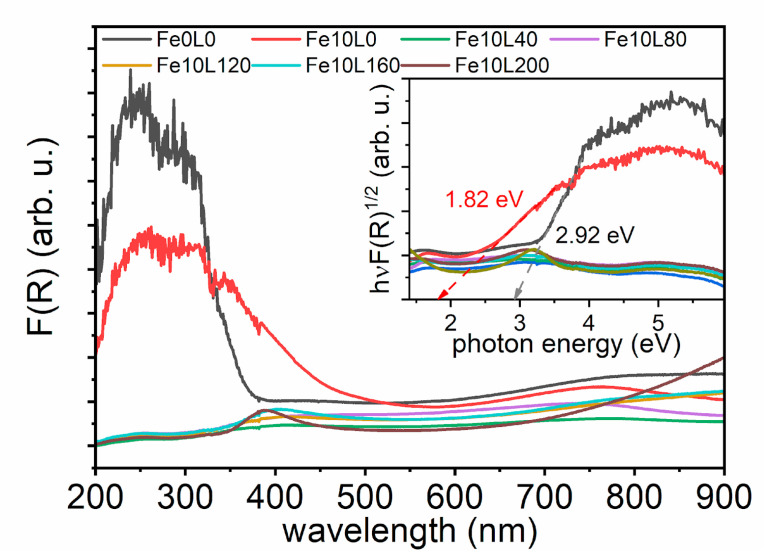
Absorbance spectra and Tauc plots (inset) for laser-treated Fe-decorated TiO_2_-NTs.

The optical properties of samples were examined by diffuse reflectance spectroscopy. The Kubelka-Munk function (F(R)) as a wavelength function is shown in Figure 8. As seen, the laser treatment with the fluence of 40 mJ/cm^2^ and higher drastically reduced absorption in the UV range. As a consequence, the determination of optical bandgap (BG) becomes possible only for Fe10L0 and Fe0L0 samples. Their values were determined based on the Tauc plot for indirect allowed transition as 1.82 and 2.92 eV, respectively. The pristine anatase, rutile, hematite, and magnetite are semiconductors with the bandgap values of 3.2, 3.0, 2.2, and 0.1 eV, respectively [62,63], while phases composed of Fe_2_O_3_-TiO_2_ [64,65,66] are expected to exhibit BG in the range of 1.5–1.9 eV. Therefore, the observed optical bandgap of Fe10L0 sample can be ascribed to the presence of non-stoichiometric or the doped hematite.

**Figure 9:**
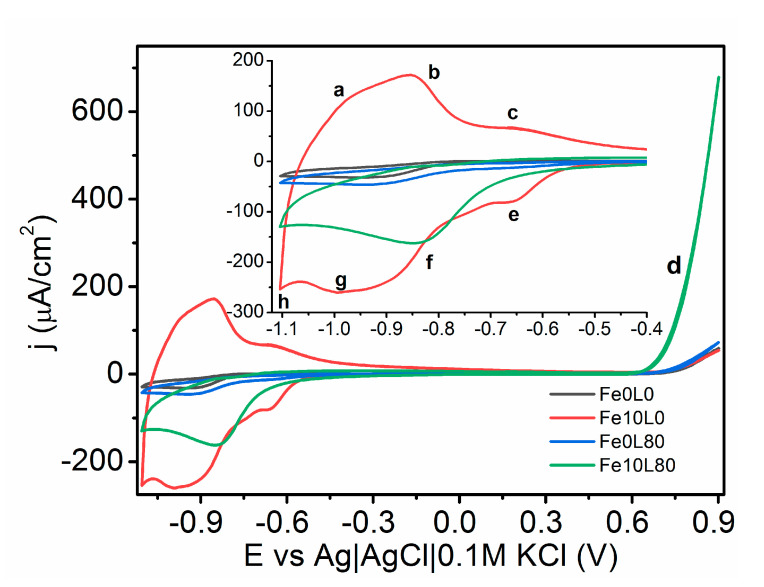
Cyclic voltammetry recorded for pristine and laser-modified; (80 mJ/cm^2^) TiO_2_-NTs in 0.5 M NaOH electrolyte in darkness.

Cyclic voltammetry scans of the unmodified and laser-annealed Fe-decorated TiO_2_-NTs with the fluence of 80 mJ/cm^2^ are shown in Figure 9. All TiO_2_-NTs exhibit very low current in the potential range from −0.45 to +0.6 V vs. Ag|AgCl|0.1 M KCl that reflects low capacitance related with low conductivity of titania and/or low surface area since we modify here the nanotubes of length below 1.6 µm. This current originates from charging/discharging of electric double layer. However, above this potential regime, oxygen evolution reaction (OER) was identified (marked as d, in Figure 9), while, below −0.45 V, the redox reactions are observed. Pairs of current peaks at −0.66 V(e)/−0.66 V(c), −0.85 V (f)/−0.85 V (b), and −0.99 V (g)/−0.96 V (a), versus Ag|AgCl|0.1 M KCl, could be ascribed to the redox reactions of 3Fe_2_O_3_ + 2e^−^ ↔ 2Fe_3_O_4_ + O^2−^, TiO_2_ + 2H_2_O + e^−^ ↔ Ti(OH)_3_ + OH^−^, and Fe_3_O_4_ + 2H_3_O^+^ + 2e^−^ ↔ 3Fe(OH)_2_, respectively [67,68,69,70,71]. The redox pair (f/b) may also correspond to the oxidation and reduction reactions of the ions in the electrolyte with the TiO_2_ film, and one possible reaction is xNa + yTiO_2_ +e^−^ ↔ Nax(TiO_2_)y [72]. According to the Pourbaix diagram, any other forms of Ti in those pH conditions are not stable in the electrochemical system. The cathodic activity initiated at −1.1 V (h) and observed for Fe10L80 and Fe10L0 samples can be ascribed to the hydrogen evolution reaction (HER).

**Figure 10:**
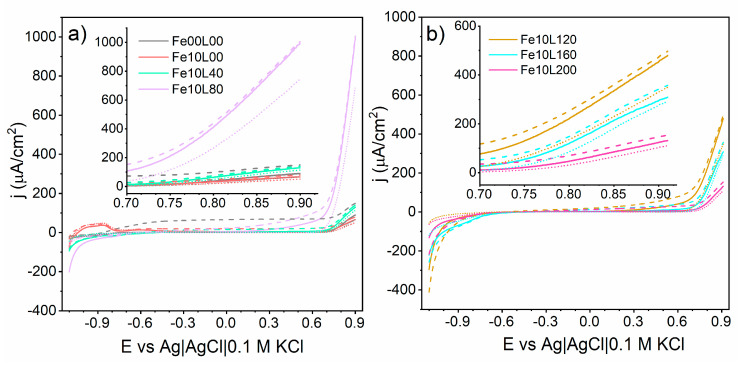
Records of linear voltammetry carried out in darkness (dotted), under simulated solar (dashed), and visible (solid) light for (**a**) bare titania and with Fe film further treated with 40 and 80 mJ/cm^2^ and (**b**) 120,160, and 200 mJ/cm^2^.

The comparison of the electrochemical performance of measured samples was carried out by the linear voltammetry technique performed in dark, under visible and full solar spectrum illumination, see Figure 10. In the potential range from −0.5 to + 0.7 V versus Ag|AgCl|0.1 M KCl the registered current of bare TiO_2_-NTs is almost constant and highly sensitive for UV light. On the other hand, activity of Fe-decorated TiO_2_-NTs samples is very low and almost light-independent until the polarization potential exceeds +0.1 V. Above this limit, the difference between registered current in dark and under light growths linearly up to beginning of OER, exceeding the photoactivity of bare TiO_2_-NTs at +0.47, +0.56, and +0.75 V by Fe10L80, Fe10L120, and Fe10L160 samples, respectively. As shown in Figure 10, the current originating from OER in dark at +0.9 V versus Ag|AgCl|0.1 M KCl grows with the applied laser fluence from 50 μA/cm^2^ for Fe10L0 to 744 μA/cm^2^ for Fe10L80 and then decreases to 101 μA/cm^2^ for Fe10L200. However, when the electrode is irradiated by visible light, the currents at +0.9 V grow up to 67, 995, and 128 μA/cm^2^, for samples from Fe10 serie: un-annealed and treated with 10 and 80 mJ/cm^2^, respectively. Only a few percent increments of registered currents were registered under UV-visible in reference to the results obtained under the visible radiation. Since the light wavelengths below 420 nm were blocked by the optical filter, the visible part of the spectrum has to be vastly responsible for the charge carriers’ creation. The highest activity of Fe10L80 in dark compared with its relatively low light absorption ability leads to the conclusion that the light conversion efficiency is the main factor limiting the electrode performance, and the well-suited laser annealing can be employed to overcome that shortage. A presence of current peaks between −0.8 V and −1.1 V is seen only for Fe10L0 sample, supporting the CV conclusion about its prevailing derivation from iron oxidation reactions. It is worth noting that the presence of 10 nm Fe on the TiO_2_-NTs leads to the photocurrent reduction under UV-visible light recorded for Fe10L0 and Fe10L40 samples (Figure 8), which shows that the presence of iron reduces the capability of electrodes to effectively transform light to charge carrier.

**Figure 11:**
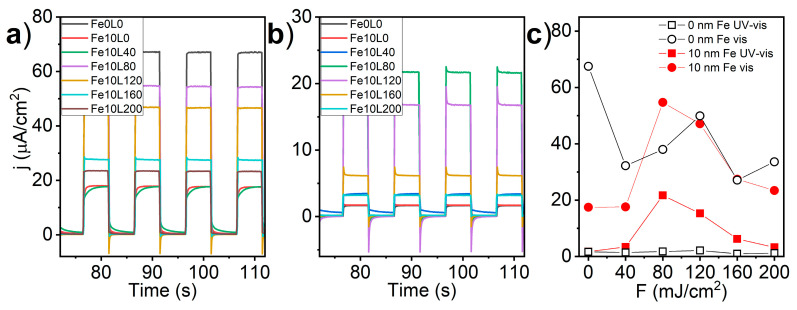
Transient photocurrents recorded for the bare and laser-annealed Fe-decorated TiO_2_-NTs in 0.5 M NaOH electrolyte under the light of full solar (**a**) and limited to visible (**b**) spectrum. Light-on current plateau of chronoamperometry records in UV-vis (circle) and visible light (square) are shown in graph (**c**).

The stability of the laser-annealed Fe-decorated TiO_2_-NTs was verified by the chronoamperometry during exposition to the chopped UV-visible and visible light registered at +0.4 V versus Ag|AgCl|0.1 M KCl. As shown in Figure 11a, the highest photocurrent density under UV-visible light was recorded for the bare sample (69 μA/cm^2^), significantly dropped for Fe10L0 and Fe10L40 samples (18 μA/cm^2^), and reached a local maximum for Fe10L80 (55 μA/cm^2^) sample. The fluence of laser annealing above 80 mJ/cm^2^ decreased the recorded currents. On the other hand, when the UV light was cut-off (Figure 11b), the highest photocurrent was registered for the Fe10L80 sample (22 μA/cm^2^), while *j* of bare TiO_2_-NTs reduces to 1.7 μA/cm^2^. As shown in Figure 11c, the laser annealing of the bare TiO_2_-NTs with a fluence of 40 mJ/cm^2^ reduces registered photocurrent under UV-visible light, while the usage of the laser of 80 and 120 mJ/cm^2^ shows local maximum of photoacitivy, similar to Fe-decorated samples. However, examination under exclusively visible light clearly shows that the Fe-decoration before laser processing is crucial to shift electrode photoactivity to the visible light.

The shape of the transient photocurrent curves under UV-vis illumination resembles the ideal square response only for three samples, namely Fe0L0, Fe10L80, and Fe10L200 (Figure 11a). In the case of Fe10L120 and Fe10L160 samples, upon illumination, the photocurrent rises instantly, but, when light is off, the photocurrent for a moment reverse the flow direction and stabilize at a few μA/cm^2^. On the other hand, Fe10L0 and Fe10L40 samples, instead of current spikes, exhibit slowly asymptotically growing/decaying signal depending on the illumination variation. Relaxation current denotes a high density of surface states mostly attributed to the surface crystalline defects and oxygen vacancies acting as recombination centers for photo-generated carriers [73,74]. Thus, variation of electrodes behavior under solar irradiation highlights the differences in the electronic structure, especially between Fe10L40 and Fe10L80 samples.

**Figure 12:**
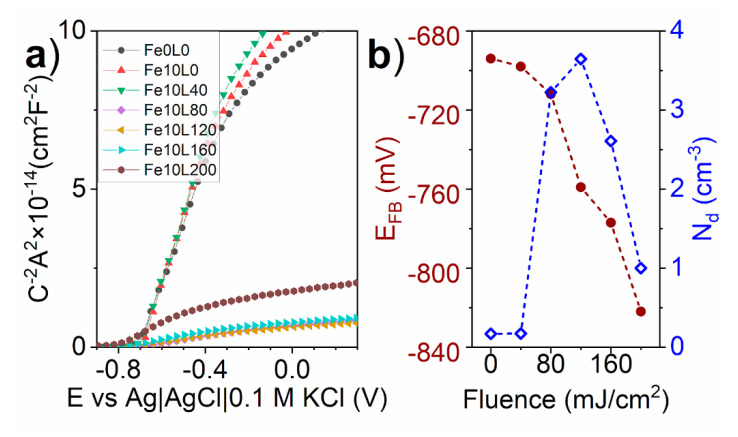
Mott-Schottky plots for bare and laser-annealed Fe-decorated TiO_2_-NTs (**a**), and E_FB_ and N_d_ as a function of laser fluence (**b**).

In order to investigate in details, the influence of the laser annealing on the charge carrier density and band position of iron decorated TiO_2_-NTs, Mott-Schottky analysis was performed. Taking into account Equation (1), the N_d_ and E_FB_ were calculated from the linear part of the Mott-Shottky plot. As shown in Figure 12, thin layer of iron causes only a minor variation in TiO_2_-NTs electrical properties, whenever the laser annealing cathodically shifts E_FB_ from −694 mV for Fe10L0 to −822 mV versus Ag|AgCl|0.1 M KCl for Fe10L200. On the other hand, N_d_ increases over 20 times from 1.71 × 10^19^ cm^−3^ for Fe10F0 to 36.4 × 10^19^ cm^−3^ for Fe10L120 but decreases for higher laser fluence. It is worthy of note that the most prominent change of N_d_ value takes place between Fe10L40 and Fe10L80 samples, showing the presence of processing threshold. That result corresponds with the XRD and Raman spectra variation between those materials, showing that the Fe10L80 sample exhibits the strongest structure distortion accompanied by oxygen vacancies formation. For a comparison, Haryński et al. [39] observed donor density increment from 2.2 × 10^19^ to 14.1 × 10^20^ cm^−3^ caused by the laser annealing using a fluence of 40 mJ/cm^2^, while Xu et al. [16] observed from 3.9 × 10^19^ to 2.66 × 10^20^ cm^−3^ using 300 mJ/cm^2^ but with KrF laser. In both cases, the authors modified TiO_2_-NTs without any former deposition of other metal/metal oxide film. Thus, it is clear that the laser interaction with titania is mostly responsible for the charge carriers’ concentration within the re-solidified layer.

It should be highlighted that the highest N_d_ was determined for the samples exhibiting most prominent transient current spikes (Figure 11b), both cathodic and anodic, under exposition to visible light. Since the capacitance of the double layer increases with the charge carriers density in the electrode, the presence of current spikes can be explained in terms of the Randles equivalent circuit of electrode-electrolyte interphase [75]. Turning on illumination causes the rapid growth of the charge carriers’ density near the double layer followed by the increment of the junction capacitance (Equation (1)). Since the C_SC_ increases almost instantly, the additional charging current associated with accumulation of holes in the electrode space charge layer is observed as an anodic spike. However, when the light is turned off, the C_SC_ decreases and causes discharging current flowing in the opposite direction. The current originated from recombination of bulk electrons with holes accumulated in the space charge layer could inflict the potential even higher than the electrode bias potential; thus, the equipment records the unloading current as a reduction spike. It is worthy of note, that the ratio of discharging to the charging spikes is proportional to the hole transfer barrier across the electrode/electrolyte junction [76]. Therefore, the recorded transient photocurrent, especially for Fe10L120 and Fe10L80 (Figure 11), implies the reduction of the hole transfer effectiveness with increasing laser fluence.

Although the Fe10L120 sample exhibits the highest N_d_, the Fe10L80 was measured to have the best electrochemical performance under the visible light. To explain that difference, the impact of the flat-band potential has to be taken into account. TiO_2_-NTs are n-type semiconductor in which the Fermi level is higher than those of the water oxidation reaction in 0.5 NaOH electrolyte. Therefore, after immersion of the TiO_2_-NTs in the electrolyte, it is starting to reach equilibrium between the Fermi levels giving a contribution to the band bending [77]. In general, the higher band bending the stronger transfer of holes towards the electrolyte, hampering their recombination with electrons. As a consequence, the negative shift of E_FB_ induced by the laser annealing reduces the band-bending and consequently reduces the oxidation under anodic but enhances reduction processes under cathodic polarization [78].

Another study of laser processing of TiO_2_-NTs has shown that, if the laser annealing is carried out in vacuum [39], it leads to the positive shift of the E_FB_, but it leads to the negative if the process was performed in water. On the other hand, it is known that the presence of hematite on the surface of TiO_2_-NTs [79], as well as their doping by Fe, leads to the negative shift of the E_FB_ [80,81]. Taking into account the variation of E_FB_ as a laser fluence function (Figure 12), it should be concluded that the iron incorporation into the TiO_2_ structure is responsible for the E_FB_ negative shift.

## 4. Conclusions

The work was focused onto the introduction of significant changes in morphology and structure of metal decorated titania via the well optimized laser treatment. Regarding the surface appearance, it has been shown that 1500 nm long TiO_2_-NTs under the laser irradiation undergo partial melting and re-solidification leading to the formation of a continuous layer hiding the ordered tubes. The combination of anatase TiO_2_, rutile TiO_2_, and a hematite Fe_2_O_3_ has been revealed. However, anatase and rutile structures were found to be distorted due to the laser interaction. Additionally, a drastic decrement of UV light absorption leading to the disappearance of the optical bandgap of samples treated with the laser of fluence equal to or higher than 40 mJ/cm^2^ was observed.

The presence of the processing threshold between samples Fe10L40 and Fe10L80 was confirmed by the rapid change in recorded transient photocurrent and the concentration of donor density, while, in the case of flat-band potential value, the cathodic shift was registered. It was proven that the charge carriers’ density was strongly enhanced by the laser processing of the TiO_2_-NTs but only up to laser fluence of 120 mJ/cm^2^. Finally, the electrochemical performance which is determined here by the charge carriers’ density, the position of Fermi level and light harvesting was found to be the highest for Fe10L80 sample. For that material the photocurrents recorded under visible light were over 20 times higher than for the bare TiO_2_-NTs. The present study has shown that the photoactivity of TiO_2_-NTs electrode can be strongly enhanced by the Fe decoration and laser annealing under optimized conditions. This successful modification begins a new approach in laser assisted synthesis of decorated by transition metal TiO_2_-NTs without the need to use any metal precursor in liquid form minimizing negative environmental impact.

Taking into account obtained results, it can be concluded that prepared samples can be used in photo-driven processes, e.g., in pollutant degradation or electrochemical water splitting enhanced by light. Moreover, Fe-TiO_2_ nanotubes are also expected to be a promising alternative for cathodic protection of metals both under dark conditions and in visible light irradiation. It should be also mentioned that Fe species not only enhance the activity of titania nanotubes but also their presence can lead to good magnetic response under applied magnetic field. Therefore, such material can find application in biomedicine, e.g., when magnetically guidable systems are required. The range of possible application is very broad, and the fabrication route proposed here can definitely help with material production not only in laboratory but also in commercial scale.
